# A Siamese Neural Network for Non-Invasive Baggage Re-Identification

**DOI:** 10.3390/jimaging6110126

**Published:** 2020-11-20

**Authors:** Pier Luigi Mazzeo, Christian Libetta, Paolo Spagnolo, Cosimo Distante

**Affiliations:** 1 Institute of Applied Sciences and Intelligent Systems—CNR, Via Monteroni sn, 73100 Lecce, Italy; pierluigi.mazzeo@cnr.it (P.L.M.); cosimo.distante@cnr.it (C.D.); 2 Department of Innovation Engineering, Campus Ecoteckne, Università del Salento, Via Monteroni, 73100 Lecce, Italy; christian.libetta@unisalento.it

**Keywords:** deep learning, Siamese Neural Networks, baggage re-identification

## Abstract

Baggage travelling on a conveyor belt in the sterile area (the rear collector located after the check-in counters) often gets stuck due to traffic jams, mainly caused by incorrect entries from the check-in counters on the collector belt. Using suitcase appearance captured on the Baggage Handling System (BHS) and airport checkpoints and their re-identification allows for us to handle baggage safer and faster. In this paper, we propose a Siamese Neural Network-based model that is able to estimate the baggage similarity: given a set of training images of the same suitcase (taken in different conditions), the network predicts whether the two input images belong to the same baggage identity. The proposed network learns discriminative features in order to measure the similarity among two different images of the same baggage identity. It can be easily applied on different pre-trained backbones. We demonstrate our model in a publicly available suitcase dataset that outperforms the leading latest state-of-the-art architecture in terms of accuracy.

## 1. Introduction

In a society where airport efficiency and security are increasingly crucial, it is strategic to invest in methodologies that can improve Baggage Handling Systems (BHS) performances. The checked-in suitcase is loaded from high speed BHS for quick processing and the stowing of it. Currently, almost all airports use X-ray technology to manage baggage: at check-in, a X-ray image of each suitcase is produced and inspected. Suspected (or in general interested) baggage is marked by means of a RFID (Radio Frequency Identification) tag. It will facilitate a security check at claim area (because an alert/alarm will be produced when the marked suitcases cross the RFID detection zone). However, a similar approach that is based on RFID technology can fail when:

- tags accidentally fall off during check-in/check-out procedures, and/or transfer process;
- passengers deliberately remove tags; and,
- in presence of metallic baggage: in this case, the RFID technology could work in a wrong way producing false positives and/or false negatives.

**Figure 1:**
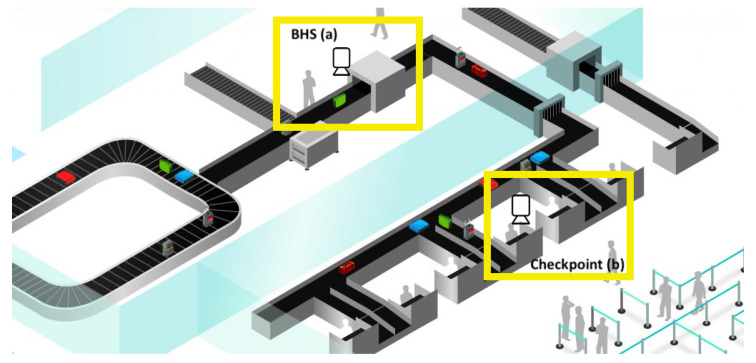
Multi-view camera system at: (**a**) Baggage Handling Systems (BHS), (**b**) checkpoint.

Moreover, the human presence is mandatory for tagging interested baggage (and it results in longer and expensive baggage sorting). All of these aspects lead to the main final drawback of this approach: the detection of interested baggage will fail, with negative consequences in terms of people security. For all of the aforementioned reasons, an approach for baggage monitoring that does not require physical tags can improve airport processes in terms of security, time, costs, efficiency. To do it, a relationship between the image scanned at BHS and related inspection information has to be created (see Figure 1). The images acquired at BHS (Figure 1a) are compared with images of baggage at the customs checkpoint (see Figure 1b). When a suspect baggage is detected, an alert can be sent to security agents and a physical check of baggage can be done by specialized agents. It should be noted that the identification of baggage can be based not only on the generic aspect of it, but also additional items can be used (stickers, tags, labels, ropes, defects—such as missing wheel, and so on).

The problem of image association among the baggage images captured at BHS (see Figure 1a) and the ones grabbed at the checkpoint represents a large research field well known as object re-identification.

The object re-identification problem is currently one of the most studied topics in the Computer Vision community with many contributions on “Person Re-identification”, “Vehicle Re-identification”, and so on. The Artificial Intelligence (AI) in recent years—with the advent of the Deep Learning—has proposed many algorithms and network architectures to solve the object re-identification problem (see Section 2). This paper proposes a siamese CNN network that is able to evaluate a baggage similarity score in order to verify whether the baggage images belong to the same identity. We take advantage of the annotated data in terms of image group similarity and image identities. During experimental session, the final convolutional activations are extracted for the Euclidean distance based suitcase similarity rate. To summarize, our contributions are: (i) we propose a siamese network that giving as input two baggage images returns their similarity measure. This network simultaneously learns a discriminative feature embedding from different CNN backbones and a similarity metric, thus improving baggage retrieval accuracy; and, (ii) we report competitive accuracy as compared to the state-of-the- art methods on the only publicly available large-scale dataset for baggage re-ID (Multi View Baggage (MVB)) [1] demonstrating that the proposed implementation outperforms them (about 20%).

The remainder of this paper is organized, as follows. Section 2 contains a review of the state of the art. Section 3 presents the proposed system. In Section 4, the experimental results obtained on a publicly available dataset are reported. Section 5 reports the conclusions.

## 2. Related Work

Re-identification (Re-ID) is the problem of identifying objects across images that have been taken while using different (overlapped or not overlapped) cameras or across time using a single camera. Re-identification is a very powerful method for video security systems as well as human computer interaction systems.

Traditional approaches have focused on low-level features such as colours, shapes, contours, and local descriptors, but with the development of deep learning, the convolutional neural networks (CNN) have dominated this field. Traditional approaches are based on discriminative classifiers, with the objective of distinguishing between the target and surrounding environment. To cope with natural image changes, these classifiers are typically trained with translated and/or scaled sample patches (often used for long term visual tracking). Kernelized Correlation Filter [2] with Histogram of Oriented Gradients (HOG) features instead of raw pixels outperforms by a large margin to other trackers, such as Struck [3] or Track-Learn-Detect (TLD) [4], working at hundreds of fps.

With the advent of the Deep learning era, new techniques that use deep learning technology, such as convolutional neural network, recurrent neural network, and generative adversarial network, are introduced to address the object ReID issue. Traditionally, the most important field of application is people PeID (based on facial landmarks, gait, posture, ...), but there are also other different applications, such as boats [5] and cars ReID [6,7].

A robust approach that integrates CNN and traditional feature detectors is proposed in [8]: here, the authors introduce a fusion feature network (FFN), which combines a variety of hand-crafted features (texture and colour histogram features) and CNN features.

In [9], the authors introduce a hybrid deep architecture for person ReID with the dual goal of reducing the variations of the intra-personal and increasing the differences of inter-personal. Another interesting work regarding the reduction of the intra-class variance for face verification task by the introduction of center loss has been proposed in [10]. Person re-identification is also the topic of the approach presented in [11]. Here, a CNN is used for learning generic deep features descriptors by combining multiple datasets together in order to train the designed CNN; in addition, a domain guide dropout strategy is introduced in order to discard worthless neurons for each domain dataset to keep the deep model in the right track.

The approach proposed that is in [12] is an evolution of [10]. Here again center loss is used for the reduction of the intra-class variation; but authors now combine it with identification loss to jointly train the designed network, also adding a feature re-weighting (FRW) layer to learn the weight coefficients of each dimension of the deep descriptors; the criticism of this approach is the over-fitting that seems to affect it.

A different point of view on re-identification problem is given by the verification model: approaches of this category take a pair of images as input and produce a similarity value in order to determine whether the paired images are the same object or not; in other words, the ReID is treated as a binary-class classification problem. An exhaustive overview on this aspect is proposed in [13].

Person Re-identification (PReID) is the core of the approach that is presented in [14]. The authors introduce a filter pairing neural network (FPNN), which includes max-out pooling layers and patch-matching; the goal is to jointly handle geometric transforms and photometric, misalignment, background clutter and occlusions. In [15], the authors introduce a “Siamese” deep network for metric learning; the proposed architecture implements three shared parameters independent convolutional networks that perform on three non-overlapping parts of the two images. The output of the fully connected layers are the deep descriptors of the two input images, and the similarity score is obtained by the evaluation of the cosine function of two output descriptors. In a similar way, in [16], the authors proposed a PersonNet model for person ReID. This work is a direct consequence of the approach that was proposed in [17] and it uses the patch matching layer to detect local relationship between patches.

The drawback of these approaches is the relatively shallow depths of their networks which do not benefit of digging the deep features to improve discrimination.

In the specific field of PReID excellent results have been obtained by combining the identification and verification models. One of the first works on this has been proposed in [18], with the use of verification and identification losses to train the network for face recognition. Later, in [19], the authors proposed a verification-identification model that combines the verification and identification losses to train the CaffeNet for person ReID.

The goal of distance metric-based deep models [13] is to reduce, as much as possible, the distances between the same person images, and increase as much as possible the distances between different person images. Several papers have followed this trend topic. Firstly, a similar model was introduced by authors in [20] to target the image retrieval task; the application of this model in the face recognition task was proposed in [21], and then, in [22], the authors first adopted the triplet model in order to address the person ReID task. The most recent state-of-the-art work on person ReID is based on the part-based deep model [13], with the goal of learning discriminative deep features. Local visual cues are close to the behaviour of human visual system and they are complementary to global information. In the field of person ReID, the combination of local and global features seems to be a good choice.

The use of hand-crafted features could seem far from the human appearance and particularly to how appearance varies between people [23]. Another interesting aspect that is related to person ReID is the dataset construction and availability of training images. Often, the number of images for one person in a certain dataset is limited in person ReID community, as highlighted in [13]. In [24], the authors first introduce a generative adversarial network (GAN) to generate unlabeled pedestrian samples and adopt a CNN sub-model for feature representation learning.

In the last years, several works on Siamese Neural Networks and their application in the field of re-identification have been produced. Siamese neural network is a kind of neural network architecture that contains two or more identical (Siamese) sub-networks; the word ’identical’ means that the sub-networks share the same architecture and the same parameters and weights for each other.

Typically, a Siamese network is employed as pairwise, with two sub-networks included. The output of Siamese model is basically a similarity score that takes place at the top of the network [25]. The last layer represents two functions: connection function, which is used to evaluate the relationship between two samples, and cost function, which is used to convert the relationship into a cost. How to choose these functions is closely related to the CNN performance [26].

## 3. Proposed System

**Figure 2:**
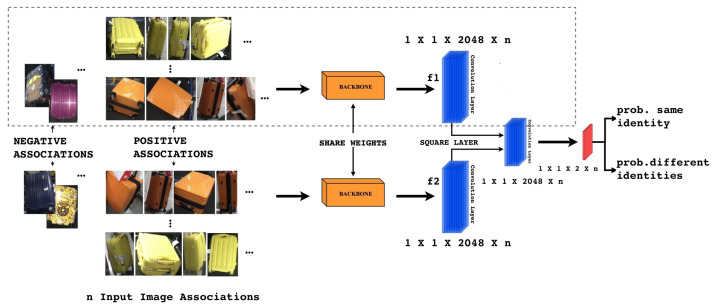
The proposed system. Given n groups of suitcase image associations, two identical Backbone models are used as the non-linear embedding functions and output 2048-dim embeddings f1, f2. Then, f1 and f2 are used to predict the identity of the input suitcase. We introduce a non-parametric layer, called Square Layer, to compare high level features f1 and f2. Finally, the softmax loss is applied on the objective.

The proposed network model is, basically, a convolutional siamese network that will be trained to determine whether or not two objects are the same or different. The goal is to associate, using a similarity loss, image inputs that belong to same identity, minimizing the distance between them and maximizing the difference between dissimilar ones. Figure 2 briefly describes the network structure. Given as input, to the backbone network, a pair of resized images the proposed siamese network, estimates the images similarity score. The network consists of two CNN backbones already pre-trained on ImageNet dataset, three convolutional layers, a square layer, and a similarity loss, as can be noticed in Figure 2. The pre-trained backbones will be ResNet-50 [27], SENet [28], and EfficientNet [29], where we have removed last fully-connected (FC) layer. The optimization objective is reached by computing a similarity loss.

### 3.1. Network Fine-Tuning

From Figure 2, it can be noticed that there are two backbones that share weights in order to predict the identities of the input image couples at the same time. We fine-tune the network on the MVB dataset (see Section 4.1), replacing the fully connected layer of the pre-trained backbone with a new *conv-layer*.

### 3.2. Similarity Loss

A non-parametric layer named Square Layer is introduced to compare the extracted high-level features $f_{1}$ and $f_{2}$, as shown in Figure 2. These features coming from the fine-tuned CNN have a discriminating capability and they are easier to handle than activation function in the intermediate levels. As input, it takes two tensors giving as outputs one tensor obtained by subtracting and squaring element-wise. This Square Layer is defined as $f_{s}={\left(f_{1}-f_{2}\right)}^{2}$, where $f_{1}$ and $f_{2}$ are the 2048-dim tensors and $f_{s}$ is the 2048-dim output tensor.

Subsequently, we add a convolutional layer and the softmax output function in order to embed the resulting tensor $f_{s}$ to a 2-dim vector $\left(p_{1},p_{2}\right)$, which is the estimated probability of the two input images that belong to the same identity (note that $p_{1}+p_{2}=1$). The third convolutional layer has $f_{s}$ as input filtering it with two kernels of size 1×1×2048. We do not add a ReLU after this convolution, and we manage baggage similarity as a binary classification problem using a cross-entropy loss, which is:

(1) $$p=softmax(\Gamma_{S}\circ f_{s})$$

(2) $$Similarity\left(f_{1},f_{2},s,\Gamma_{S}\right)=\sum_{i=1}^{2}-p_{i}\log p_{i}$$

where $f_{1},f_{2}$ are the two tensors of of size 1×1×2048 containing the high-level features; *s* is the binary target class (same/different identity), $\Gamma_{S}$ represents the added convolutional layer parameters, and *p* is the inferred probability. If the image pair contains the same luggage, then $p_{1}=1,p_{2}=0$, if not, $p_{1}=0,p_{2}=1$.

### 3.3. Backbone

Convolutional neural networks represent a more than valid alternative to traditional methods in various computer vision tasks, such as classification, segmentation, and so on. Over the years, increasingly deep and complex networks have been developed in order to improve their performance. Usually a pre-trained model is used and its performance is improved by performing a fine-tuning operation or new levels or improved techniques are used to make the network deeper. Many of the works that are seen in Section 2 use the VGG-16 network as a backbone, in the proposed architecture different backbones have been used to try to achieve better performance. As previously said, we test three different pre-trained networks as backbones in the proposed architecture; in the following subsections, they will be briefly presented.

#### 3.3.1. ResNet

**Figure 3:**
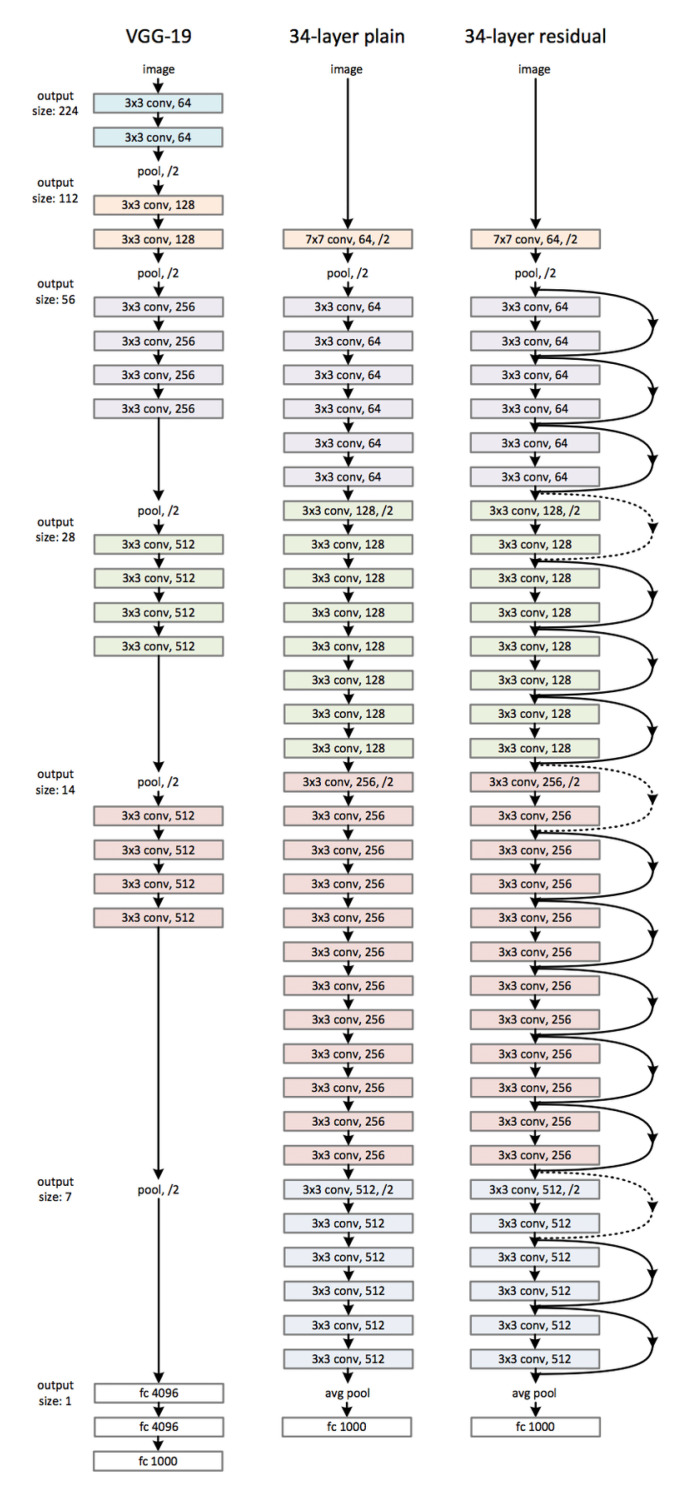
(**Left**): the VGG-19 model as a reference. (**Middle**): a plain net- work with 34 parameter layers. (**Right**): a residual network with 34 parameter layers [27].

The architecture of the ResNet, as specified in [27], is mainly based on that of the VGG network (Figure 3-left), in which the convolutional layers mostly have 3 × 3 filters and follow two simple design rules:

- for the same output feature map size, the layers have the same number of filters; and,
- if the feature map size is halved, the number of filters is doubled, so as to preserve the time complexity per layer.

Downsampling is directly performed by convolutional layers that have a stride of 2. The network ends with a global average pooling layer and a 1000- way fully-connected layer with softmax. The total number of weighted layers is 34 (Figure 3-middle). When considering the aforementioned network, then there are inserted shortcut connections (Figure 3-right), which turn the network into its counterpart residual version. The identity shortcuts can be directly used when the input and output are of the same dimensions. When the dimensions increase (dotted line shortcuts in Figure 2), we consider two options:

1. the shortcut still performs identity mapping, with extra zero entries padded for increasing dimensions. This option introduces no extra parameter; and,
2. the projection shortcut is used to match dimensions (done by 1×1 convolutions).

For both options, when the shortcuts go across feature maps of two sizes, they are performed with a stride of 2.

#### 3.3.2. SENet

**Figure 4:**
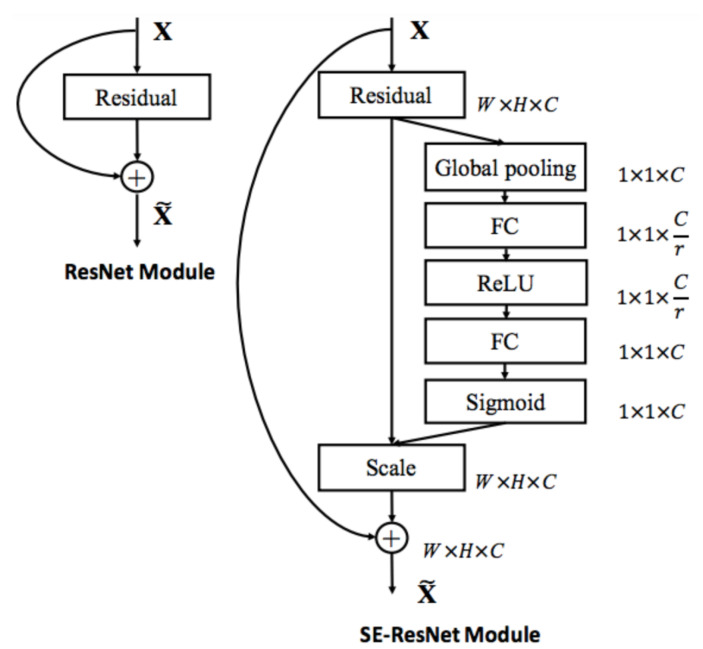
The schema of the original Residual module (**left**) and the SE- ResNet module (**right**) [28].

Squeeze-and-Excitation Networks (SENets) [28] introduce a building block for CNNs that improves channel interdependencies at almost no computational cost and they helped to improve the result by 25%. Besides this huge performance boost, they can be easily added to existing architectures. The main idea is: “Let’s add parameters to each channel of a convolutional block so that the network can adaptively adjust the weighting of each feature map.” As simple as it may sound, this is it. So, let us take a closer look at why this works so well and how we can potentially improve any model with this network. CNNs use convolutional filters in order to extract hierarchical information from images. Lower layers find trivial pieces of context, like edges or high frequencies, while upper layers can detect faces, text or other complex geometrical shapes. They extract whatever is necessary to solve a task efficiently. All of this works by fusing the spatial and channel information of an image. The different filters will first find spatial features in each input channel before adding the information across all available output channels. The network weights each of its channels equally when creating the output feature maps. SENets are all about changing this by adding a content aware mechanism to weight each channel adaptively. In it is most basic form, this could mean adding a single parameter to each channel and giving it a linear scalar how relevant each one is. In Figure 4, we can see an SE block:

1. the function is given an input convolutional block and the current number of channels it has;
2. we squeeze each channel to a single numeric value using average pooling;
3. A fully connected layer followed by a ReLU function adds the necessary nonlinearity. Its output channel complexity is also reduced by a certain ratio;
4. a second fully connected layer followed by a Sigmoid activation gives each channel a smooth gating function; and,
5. at last, we weight each feature map of the convolutional block based on the result of our side network.

These five steps add almost no additional computing cost (less than 1) and they can be added to any model.

The authors in [28] also showed that, by adding SE-blocks to ResNet-50, you can expect almost the same accuracy as ResNet-101 delivers. This is impressive for a model requiring only half of the computational costs.

#### 3.3.3. EfficientNet

**Figure 5:**
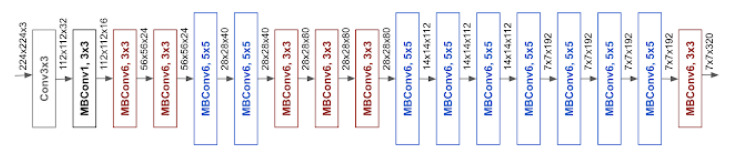
The architecture for baseline network EfficientNet-B0 is simple and clean, making it easier to scale and generalize. [29].

When compared to other models achieving similar ImageNet accuracy, EfficientNet is much smaller. For example, the ResNet50 model as you can see in some applications has 23,534,592 parameters in total, and even though, it still underperforms the smallest EfficientNet, which only takes 5,330,564 parameters in total. We will dive into its base model and building block, like for the classical ResNet model is identity and convolution block. For EfficientNet, as it can be noticed in Figure 5, its main building block is mobile inverted bottleneck MBConv, which was first introduced in MobileNetV2. By directly using shortcuts between the bottlenecks that connect a much fewer number of channels as compared to expansion layers, combined with depth-wise separable convolution that effectively reduces the computation by almost a factor of $k^{2}$, when compared to traditional layers. Where *k* stands for the kernel size, specifying the height and width of the two-dimensional (2D) convolution window.

In [29], a squeeze-and-excitation (SE) optimization is also added, which contributes to further performance improvements. The second benefit of EfficientNet is due to the fact that it scales more efficiently by carefully balancing network depth, width, and resolution, which lead to better performance.

## 4. Experimental Results

We tested the proposed algorithm on a public available dataset against the up to date state of the art methods. The network architecture used for training phase is that described in Section 3. In particular, different well-known backbones have been used: ResNet50 [27], SeNet [28], and EfficientNet-b5 [29].

### 4.1. MVB Dataset

The dataset that we used in our experiments (provided by Zhang et al. [1]), contains the largest available public collection of suitcases images. MVB Dataset contains 4519 suitcase identities (classes) and 22,660 annotated suitcase images, the train set contains 4019 suitcase identities, while the validation set contains 500 suitcase identities.

The image sizes is not unique for all; it varies from image-to-image, since all of the images are captured by specially-designed multi-view camera system in order to handle pose variation and occlusion.

**Figure 6:**
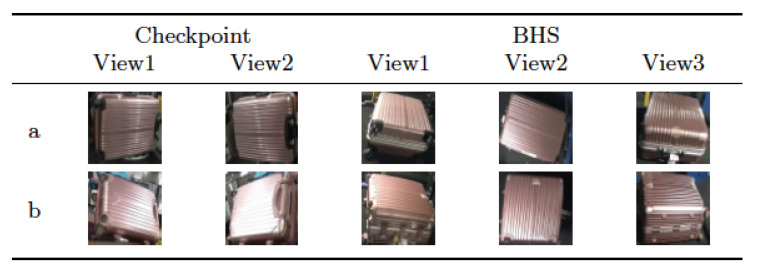
Samples of inter-class similarity on MVB (Multi View Baggage) dataset. Images in each row are from one identity. (**a**) different image views of the baggage. (**b**) different image views of the another baggage [1].

**Figure 7:**
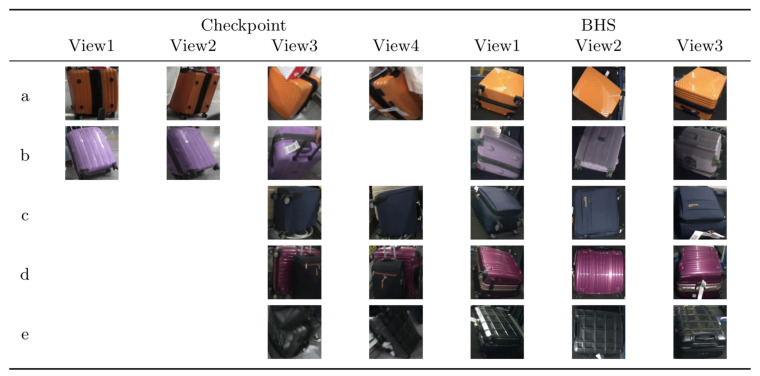
Samples of intra-class dissimilarity on MVB. Blank cell indicates corresponding view image is not valid. Images in each row represent the same identity. (**a**) images containing different viewpoint and pose; (**b**) images taken with different lighting conditions; (**c**,**d**) images with different occlusions; (**e**) images blurred for passenger motion [1].

Some suitcases are naturally very hard to distinguish from each other according to their appearance, even more difficult than the context of person Re-ID. The suitcases can look very similar, but they actually have different identities. Two suitcases that look very similar, but in real scenario have two different identities, as it can be noticed in Figure 6. The cues to distinguish them are hiding in detail of images. Meanwhile, the images of BHS and checkpoint are substantially different. The intra-class dissimilarity aspects are shown in Figure 7. It can be noticed that: (i) suitcase images have quite different backgrounds between BHS and checkpoint. Mainly black conveyor belt in BHS images, meanwhile in checkpoint images background depends on the context (i.e. passengers clothes, body parts, floor, and so on); (ii) suitcase on cart can lead to heavy occlusion in checkpoint images (Figure 7d), while the checkpoint image might be also partially invisible in BHS image, because surface is at bottom, which corresponds to the case in Figure 7c; (iii) baggages are essentially unlike, due to different locations of cameras, and a suitcase can be in various poses, such as Figure 7a showed; (iv) lighting conditions at BHS and checkpoint are not the same which often leads to color and reflection differences. For instance, Figure 7b displays obviously different color characteristics at BHS and checkpoint; and, (v) as passengers walking through checkpoint gate at different speed, motion blur makes the suitcase image to be less distinctive, as shown in Figure 7e.

All of these above factors make suitcase Re-ID on MVB dataset a challenging and inspiring task between different domains.

The MVB dataset is composed by images that were captured from seven different cameras and relevant label has been applied to each image, following very precise rules.

**Dataset and data Augmentation.** We generate the pair data for the proposed Siamese network training, from all positive pairs, i.e., pair of suitcase images that has the same identity, among 4019 identities are employed as training data; instead, negative training pairs are randomly sampled between all different identities. This way, we create a training set that is balanced in both positive and negative labels. The proposed Siamese network is then trained, with five epochs on this balanced training set. We used the trained output model to infer 500 baggage identities that were randomly taken from the 4019 identities in order to execute hard example mining: High confidence False positive pairs are selected as supplement negative pairs and then added to original training set. Furthermore, it was necessary to augment the examples in the starting dataset given the complexity of the network and the number of parameters to be trained during the fine-tuning procedure. For this reason we made the geometrical transformations (i.r. flipping, rotation), a centred and square cropping on baggage image examples, increasing the total number of pairs up to 75k. At the end of this process, the amount ratio of the positive and negative pairs in the obtained augmented training set is about 1:2. All of the resulting instance of the training set was resized to the required size of the backbone and the network input layer.

**Performance metrics.** We report the most commonly used metrics for evaluating the performance, which include: the mean average precision (mAP) and Cumulated Matching Characteristics (CMC).

CMC is adopted as evaluation metric in order to measure the performance of baggage ReID on MVB, since there is only one ground-truth identity among the gallery of 500 identities. In this paper, CMC at rank1, rank5, and rank10 will be evaluated.

mAP quantifies how good our model is at performing the query. Going deeply, the mean average precision (mAP) of a set of queries is defined as:

(3) $$mAP=\frac{\sum_{q=1}^{Q}AveP(q)}{Q}$$

where *Q* is the number of queries in the set and *AveP*(*q*) is the average precision (*AP*) for a given query, *q*. For a given query, *q*, we calculate its corresponding AP, and then the mean of all these AP scores would give us a single number, called the mAP, which quantifies how good our model is at performing the query. We have chosen to use this metric, because mAP has been widely used in this kind of applications [6,30].

### 4.2. Implementation Details

Section 3 describes the proposed network model. Training and evaluation was performed on NVIDIA TITAN RTX GPU 24GB while using the PyTorch framework.

Using a backbone network already trained for a different task allows for us to take advantage from the extracted feature boosting the whole training phase. This way, we can fine-tune our network while using different backbones already trained on large datasets.

Table 1 summarizes the different hyperparameters used during the network training phase; as it can be noticed, they depend on the used different backbones. The first column contains the input image size (*wxh*), second one the learning rate value, finally the last one, the batch size dimension. We adopt the mini-batch stochastic gradient descent (SGD) in order to update the parameters of the network.

The dataset has been splitted into four subsets: “train”, “val”, “query”, and “gallery”, in order to train and test the different network performance while using the different backbones as feature extractor.

Table 2 contains the accuracy performance for ResNet50, SENet, and EfficientNet backbones. It can be noticed that, in all three models, rewarding results are achieved with regard to the accuracy on the training and validation sets.

**Figure 8:**
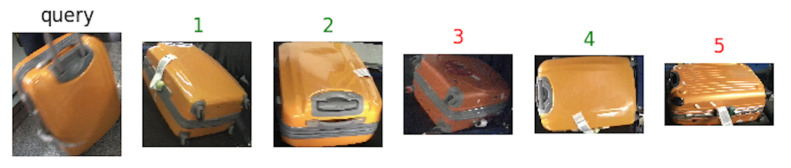
Results with Resnet50 as backbone. Green labels are correct query answers, while red ones indicate different identities.

**Figure 9:**
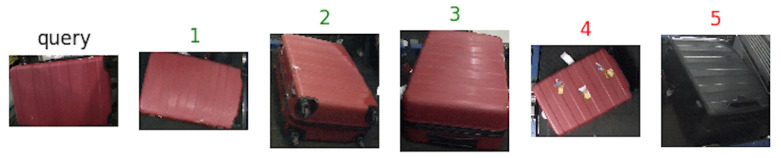
The results with EfficientNet-b5 as backbone. Green labels are correct query answers, while the red ones indicate different identities.

**Figure 10:**
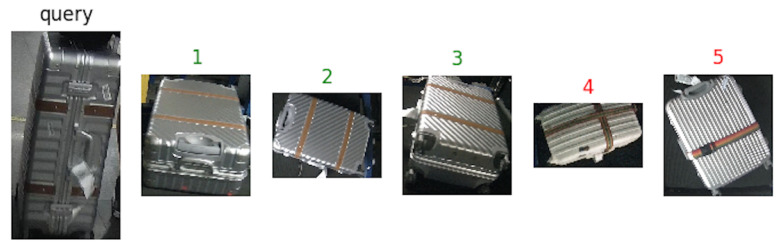
Results with with Squeeze-and-Excitation Networks (SENet) as backbone. Green labels are correct query answers, while the red ones indicate different identities.

Figure 8, Figure 9 and Figure 10 show an example of how the proposed architecture works: a “query” image is the network input (first from the left), while the other five are the network outputs, which are the most similar suitcases extracted from the gallery. The number above the images represents the rank, while the color stands for: green (same suitcase) or red (different suitcase).

The results that are contained in Table 3 clearly demonstrate that the proposed architecture with EfficientNet-b5 backbone achieves the best performance in terms of CMC rate. It can be noticed that the proposed solution outperformed the network architecure that was decribed by Zhan et al [1] in terms of Rank1 rate, on the same MVB dataset (about 20 %).

### 4.3. Comparison with Other Datasets

In order to demonstrate the reliability of the proposed approach in different contexts, it has been also tested in two different application fields, specifically boat re-identification [31] and person re-identification. With reference to the boat re-identification problem, our approach has been tested on a publicly available dataset: it is composed by 107 classes, and each class represents a different boat with a total of 5523 images. Some images have been obtained by augmenting the original dataset by means of morphological operators that were applied to original images. In Table 4, the obtained results are presented. As it can be seen, they confirm the goodness of the proposed approach, even if a comparison cannot be proposed, because this dataset is very new.

With reference to the person re-identification, we have tested our system in two different datasets for persons re-identification:

**Market1501** [32] contains 32.668 annotated bounding boxes of 1.501 identities. Images of each identity are captured by at most six cameras. According to the dataset setting, the training set contains 12,936 cropped images of 751 identities and testing set contains 19.732 cropped images of 750 identities.
**CUHK03** dataset [14] is composed by 14.097 cropped images of 1.467 identities collected in the CUHK campus. Each identity is observed by two camera views and has 4.8 images in average for each view. Following the setting of the dataset, the dataset is partitioned into a training set of 1367 persons and a testing set of 100 persons.

In Table 5, the results obtained on these dataset are presented. For completeness, we also propose the results obtained on the same dataset by the architectures proposed in [19]. As it can be seen, the proposed solution is comparable with [19], and outperforms it in the EfficientNet b-5 configuration. The encouraging results that were obtained on both of these two different application contexts confirm the usability and adaptability of the proposed system: we have obtained interesting results on boats, people and baggage.Again, the obtained results confirmed the goodness of the proposed network implementation to solve the re-identification problems.

## 5. Conclusions and Future Perspectives

This paper proposed a new Siamese Neural Network-based model able to perform an efficient baggage re-identification in an airport baggage handling system. The proposed model learns discriminative features in order to measure similarity among two different images of the same suitcase identity. We demonstrated our model in a public available suitcase dataset and it outperformed in terms of performance the leading latest state-of-the-art architecture.

The proposed re-identification model could be used as a brick in more complex monitoring systems; in this case, it should be noted that it has to be considered as a ’pure’ re-id system: its goal is the detection/validation of similarity between objects (suitcases). In other words, the overall architecture should be able to provide, as network input, image pairs: the first one, acquired at the check-in, the second one (as potential matching), acquired at some checkpoints on the BHS (for example, all of the suitcases at the check-out).

Future works will include testing of new network backbones, including geometrical and spatial features, in order to further improve the performance of suitcase re-identification on the large-scale testing set.

## Figures and Tables

**Table 1:** Training hyperparameters for different backbone

Backbone	*h* × *w*	lr	Batch Size	
ResNet50 [27]	224×224	0.05	64
SENet [28]	224×224	0.05	32
EfficientNet-b5 [29]	250×250	0.05	64

**Table 2:** Top results of proposed methods at Rank 1, 5, 10, and mean average precision (mAP) on MVB dataset.

Backbone	Rank1 (%)	Rank5 (%)	Rank10 (%)	mAP
ResNet50 [27]	64.82	85.26	90.68	61.65
SENet [28]	67.49	85.26	90.68	63.58
EfficientNet-b5 [29]	69.10	85.74	90.68	63.11

**Table 3:** Performance comparison with the solution proposed in [1].

Architecture	Rank1 (%)
**Proposed**	**69.10**
MVB [1]	50.19

**Table 4:** Performance of the three tested architectures on boat Re-id dataset in terms of mAP and Rank-1.

Method	mAP	Rank-1
RESNET50	86.78	98.85
SENet	84.91	96.64
EfficientNet-b5	89.92	98.85

**Table 5:** Comparison of the results at Rank 1 and mAP on public datasets.

	Market1501 Dataset [32]		CUHK03 Dataset [14]	
Method	Rank1(%)	mAP (%)	Rank1(%)	mAP
CNN embedding (CaffeNet [19])	62.14	39.61	59.80	65.80
CNN embedding (VGG16 [19])	70.16	47.45	71.8	76.5
CNN embedding (ResNet-50 [19])	79.51	59.87	83.4	86.4
Ours (ResNet-50)	77.13	58.42	79.8	78.2
Ours (SENet)	82.53	71.18	82.8	89.3
**Ours (EfficientNet-b5)**	**85.79**	**80.92**	**89.6**	**89.1**
